# Supplementary material for: MetaRibo-Seq measures translation in microbiomes
Source: Nat Commun. 2020 Jun 29;11:3268. doi: 10.1038/s41467-020-17081-z (PMC7324362; doi:10.1038/s41467-020-17081-z)
Supplement: Supplementary file 10 — Supplementary Data 7 [file 41467_2020_17081_MOESM10_ESM.zip › File2/Confidence_High_Taxonomy/204456_out.krona.html]

Javascript must be enabled to view this page.

members
magnitude
magnitudeUnassigned
count
unassigned
taxon
rank

204456\_out

4

superkingdom
4
2

phylum
4
1239


SRS143181\_contig\_number\_contig-100\_157.67255
2292896
species
1

class
3
186801

186802
order
3

3
family
31979

1485
3
genus


SRS016335\_contig\_number\_12514SRS047014\_contig\_number\_35312SRS098514\_contig\_number\_contig-100\_109.3984
59620
3
species
